# Modeling Parental Influence on Food Consumption among Chinese Adolescents through Self-Efficacy: A Path Analysis

**DOI:** 10.3390/nu13124454

**Published:** 2021-12-14

**Authors:** Jialin Fu, Fang Liang, Yechuang Wang, Nan Qiu, Kai Ding, Jing Zeng, Justin Brian Moore, Rui Li

**Affiliations:** 1 Department of Healthcare Management, School of Public Health, Wuhan University, Wuhan 430071, China; Fjl0708@whu.edu.cn (J.F.); fliang@whu.edu.cn (F.L.); ywang20@whu.edu.cn (Y.W.); 2013302170051@whu.edu.cn (N.Q.); 2021203050024@whu.edu.cn (K.D.); 2021283050065@whu.edu.cn (J.Z.); 2 Department of Implementation Science, Division of Public Health Sciences, Wake Forest School of Medicine, Winston-Salem, NC 27101, USA; jusmoore@wakehealth.edu; 3 School of Nursing, Wuhan University, Wuhan 430071, China

**Keywords:** parental control, parental modeling, parent–teen co-decision making, self-efficacy, food consumption, adolescent

## Abstract

This study aimed to investigate the associations between perceived parental control, perceived parental modeling and parent–teen co-decision making, and fruit and vegetable (F&V) and sugar-sweetened beverage and junk food (S&J) consumption among Chinese adolescents, and examine whether self-efficacy mediates the associations. Data were collected in a cross-sectional survey of Chinese adolescents carried out in the fall of 2019. The questionnaires were adapted from the Family Life, Activity, Sun, Health, and Eating (FLASHE) Study. Ordinary least-squares regressions and a path analysis were performed to evaluate the hypothesized associations. The final sample included 3595 Chinese adolescents (mean (SD) age, 14.67 (1.73) years; 52.82% (*n* = 1899) males). Perceived parental control was positively associated with adolescents’ F&V consumption, and was negatively associated with adolescents’ S&J consumption. Perceived parental modeling and parent–teen co-decision making were both positively associated with adolescents’ F&V consumption and negatively associated with their S&J consumption. Adolescents’ self-efficacy was positively associated with F&V consumption and negatively associated with S&J consumption. These results suggest that serving as a positive role model, having adolescents participate in the decision-making process, and increasing adolescents’ self-efficacy can be feasible and efficacious strategies to improve the nutritional quality of Chinese adolescents’ diets.

## 1. Introduction

A healthy diet characterized by sufficient intake of fruits, vegetables, whole grains, and lean proteins, and a low intake of saturated fats, sugar, and processed food products can protect against obesity and prevent chronic diseases [1,2,3]. Accumulating epidemiological evidence has demonstrated that unhealthy dietary behaviors and poor diet quality are key risk factors for various chronic diseases and mental health concerns in adolescents [1,4,5,6,7]. A growing body of research supports the assertion that diet-related problems, including obesity and eating disorders, which has long been regarded as more of a concern for Western cultures, are becoming more common among Chinese adolescents [8,9,10].

Dietary behavior is influenced by individual, social, cultural, and environmental factors [11]. Interpersonal relationships and societal norms influence food consumption behavior in adolescents [12]. Several studies have indicated that youth, and adolescents especially, are influenced by their peers’ consumption behavior [12,13,14]. In addition, parents play an important role in shaping their dietary behavior of adolescents [15,16]. Previous studies have consistently demonstrated that parental food habits and feeding strategies are the strongest determinants of an adolescent’s eating behavior and food choices [17,18,19,20]. According to Kelman, there are three broad processes of social influence: compliance, identification, and internalization [21]. The process through which humans adopt a behavior because of an increased desire to gain approval or rewards from an authority is referred to as compliance. Identification describes the process in which humans are influenced by someone who is respected or admired. Internalization is defined as an effect that occurs when humans accept a belief because they feel that it fits with their inner values [22]. Kelman’s model offers a theoretical framework that distinguishes several influence strategies that can be used to explore parental influence on adolescents’ food consumption [23]. Specifically, adolescents may choose to eat healthy foods because they desire to please their parents to receive a reward or avoid punishment. Adolescents may imitate or adopt eating behavior when they observe an influential role model in their lives (e.g., parents). Additionally, parents can operate their influence by having adolescents internalize good eating habits into their own value systems. Based upon Kelman’s model, several studies have indicated that perceived parental control corresponds to compliance, perceived parental modeling (what Kelman labeled as identification), and parents making decision together with adolescents [23].

Previous studies that have reported the associations between the three factors of Kelman’s model and adolescent’s food consumption have shown conflicting results [24]. Some studies indicated that parental control is positively associated with healthy dietary behavior [25,26,27], whereas some suggested that control can be problematic [28,29,30,31]. The data are more consistent for parental modeling, which is typically found to be positively associated with healthy food consumption in adolescents [24]. Fewer studies have looked at parent–teen co-decision making. However, internalized beliefs and values travel with people and can affect behaviors without surveillance; therefore, parent–teen co-decision making can potentially play a significant role in adolescents’ diet choices [32]. Recent research conducted in America noted that parent–teen co-decision had a positive effect on adolescents’ consumption of fruit and vegetables, but was not associated with adolescents’ sugary drink or less healthy food consumption [23]. However, no study has been conducted to date that investigates how these three influential factors are associated with Chinese adolescents’ food consumption. In addition, adolescents’ self-efficacy, as a key role player in the development of health attitudes and behavior, was found to have significant mediating effect on the association of parental influence and adolescents’ nutrition intakes. Adolescents’ increased self-efficacy for maintaining adequate healthy food intake or restricting the less healthful foods contributes significantly to their better food choices [23,33,34,35].

Therefore, in this study we examined the associations between perceived parental control, perceived parental modeling and parent–teen co-decision making, and F&V and S&J consumption in a large sample of Chinese adolescents. Additionally, we tested the mediating effects of self-efficacy in the relationships. We hypothesized that perceived parental control, perceived parental modeling and parent–teen co-decision making are positively associated with adolescents’ F&V consumption and negatively associated with adolescents’ S&J consumption, and we hypothesized that adolescents’ self-efficacy for eating F&V and limiting consumption of S&J mediates the relationships between the three perceived parental influence factors and the consumption frequency of F&V and S&J.

## 2. Methods

### 2.1. Ethics Statement

The present study was conducted in October 2019 in Wuhan, China. Participants signed informed consent forms, and participation was voluntary. Data collection procedures were conducted in accordance with the Declaration of Helsinki, and all the procedures involving human subjects were guided by the Wuhan University Ethics Board (ethical approval code: 2019YF2056).

### 2.2. Participants

**Figure 1:**
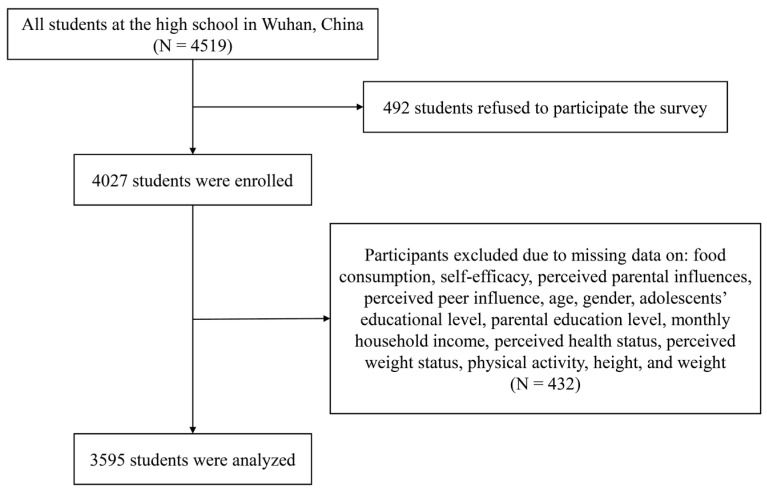
Flow chart of participants.

This cross-sectional study involved a convenience sample from a high school in Wuhan, China. On the principle of convenience sampling, all students at the high school represented eligible participants. A total of 4519 participants were invited to enroll in the study, and 89.11% of them consented to participate. However, 432 participants failed to provide sufficient identifying data and were excluded. Finally, the analytic sample was composed of 3595 participates (Figure 1).

### 2.3. Measures

Participates provided information about their age, gender, adolescents’ educational level, parental education level, monthly household income, perceived health status, perceived weight status, physical activity, height, and weight. Adolescents’ educational level was categorized as junior high school and senior high school. The highest education level for either the mother or father was used as the parental education level, which was classified as illiterate or primary school, middle school or high school, college or above. Monthly household income was divided as ≤5000 RMB, 5000–10,000 RMB, 10,000–20,000 RMB, 2000–40,000 RMB, or ≥40,000 RMB. The self-evaluation of health status was measured by asking participants “How do you feel about your current health?” using a 5-point response scale ranging from excellent (1) to poor (5). The self-perception of weight status was identified by questioning “How do you feel about your weight now?” with a 5-point response scale of very underweight (1) to very overweight (5). Physical activity was assessed by estimating minutes of moderate to vigorous physical activity (MVPA) per week according to the self-reported Youth Activity Profile [36]. BMI was calculated from self-reported height and weight.

Questionnaires from the FLASHE Study were used to collect information about the perceived parental control, perceived parental modeling, parent–teen co-decision making, perceived peer influence, self-efficacy, and the consumption frequency of F&V and S&J. The FLASHE Study was developed by the American National Cancer Institute through cognitive and usability testing [37]. Details of the FLASHE Study are available elsewhere (https://cancercontrol.cancer.gov/brp/hbrb/flashe-study (accessed on 22 September 2021)). Perceived parental control, perceived parental modeling, parent–teen co-decision making, perceived peer influence, and self-efficacy were evaluated by items on 5-point Likert scale (1 = strongly disagree; 5 = strongly agree). Consumption of F&V and S&J were computed as the daily consumption by the consumption frequency in past 7 days. Detail items and responses of perceived parental influence, self-efficacy, and food consumption could be found in the Supplementary Table S1. The questionnaire was translated into Chinese and has proven to have a good reliability and validity [38]. We assessed reliability of the questionnaires using Cronbach’s alpha coefficient and we performed exploratory factor analysis to evaluate validity: Cronbach’s Alpha_Perceived parental influence_ = 0.70; Kaiser–Meyer–Olkin = 0.71, P Bartlett < 0.001.

### 2.4. Statistical Analyses

Descriptive statistics were calculated to show baseline characteristics of the enrolled participants. The Shapiro–Wilk normality test was performed to check data normality. To quantify the correlations between three perceived parental influence factors, self-efficacy, and the consumptions frequency of F&V or S&J, spearman correlation analyses were used. Ordinary least-squares regression was used to quantify the associations of the three perceived parental influence factors and self-efficacy with the frequency of F&V or S&J consumption. To test the mediating effects of self-efficacy in the relationships between the three perceived parental influence factors and the consumption frequency of F&V and S&J, two mediation analyses were conducted using the PROCESS for SPSS developed by Hayes (2017). A bootstrap estimation approach with 5000 samples was performed to evaluate β-coefficient with 95% CI. Age, gender, adolescents’ educational level, parental education level, monthly household income, perceived health status, perceived weight status, perceived peer influence, BMI, and physical activity were included as covariates in the regression models and the mediation analyses. All statistical analyses were performed using SPSS 21.0 (IBM, Armonk, NY, USA). All p-values were two-tailed, and *p*-values < 0.05 was considered statistically significant.

## 3. Results

A total of 3595 adolescents (mean (SD) age, 14.67 (1.73)), including 1899 (52.82%) males, were enrolled in this study. Participating individuals were aged between 10 and 20 years with 99.28% (3569 of 3595 participants) between years 12 and 18, including 1632 (45.40%) were senior high school students (aged 14–20 years), 1963 (54.60%) were senior high school students (aged 10–15 years). Among them, 946 (26.31%) participants’ perceived health status were fair or poor, and 568 (15.80%) participants’ perceived weight status were very underweight or very overweight. The mean value of BMI was 21.12 ± 4.65. For physical activity, participants reported 751.05 ± 187.41 min MVPA per week (Table 1).

As shown in Table 2, there were significant correlations among perceived control, perceived modeling, parent–teen co-decision making and adolescents’ F&V consumption (*p* < 0.01). Ordinary least-squares regression result showed parental control (b = 0.28, *p* < 0.001), parental modeling (b = 0.10, *p* = 0.024), and parent–teen co-decision making (b = 0.20, *p* < 0.001) had a significant association with adolescents’ F&V consumption (Table 3).

Parental modeling (r = −0.10, *p* < 0.01; b = −0.12, *p* = 0.026) and parent–teen co-decision making (r = −0.11, *p* < 0.01; b = −0.14, *p* = 0.029) were found to be significantly negatively associated with adolescents’ S&J consumptions in spearman correlation analysis (Table 2) and ordinary least-squares regression model (Table 3). Parental control was found to be positively associated with adolescents’ S&J consumption (r = 0.05, *p* < 0.01; b = 0.19, *p* < 0.001).

**Figure 2:**
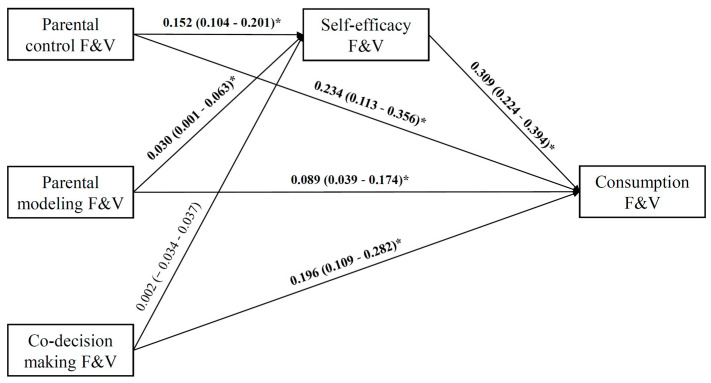
Path model of adolescents’ fruit and vegetable (F&V) consumption, adjusted for adjusted for age, gender, adolescents’ educational level, parental education level, monthly house-hold income, perceived health status, perceived weight status, perceived peer influence, BMI, and physical activity. β-coefficient and 95% CI are in parentheses. Significant results are bolded. * *p* < 0.05.

Figure 2 showed that adolescents’ self-efficacy for eating F&V significantly mediated the statistical effect of parental control (b = 0.047, 95% CI = 0.027–0.070) and parental modeling (b = 0.009, 95% CI = 0.001–0.021) on their consumption of F&V, whereas not of parent–teen co-decision making (b = 0.001, 95% CI = −0.011–0.012). Specifically, parental control and parental modeling were positively associated with adolescents’ self-efficacy for eating F&V, whereas parent–teen co-decision making showed no statistically significant connection with adolescents’ self-efficacy for eating F&V (b = 0.002, 95% CI = −0.034–0.037). Adolescents’ self-efficacy for eating F&V predicted more actual consumption (b = 0.309, 95% CI = 0.224–0.394). As to direct effects, all kinds of parental influence, were positively associated with adolescents’ F&V consumption after controlling for self-efficacy.

**Figure 3:**
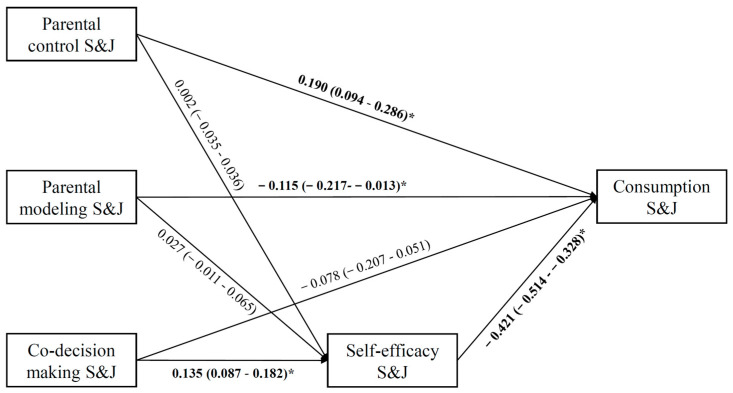
Path model of adolescents’ sugar-sweetened beverage and junk food (S&J) consumption, adjusted for adjusted for adjusted for age, gender, adolescents’ educational level, parental education level, monthly household income, perceived health status, perceived weight status, perceived peer influence, BMI, and physical activity. β-coefficient and 95% CI are in parentheses. Significant results are bolded. * *p* < 0.05.

Adolescents’ self-efficacy for limiting S&J only significantly mediated the effect of parent–teen co-decision making (b = −0.567, 95% CI = −0.084–−0.033), whereas not of parental control (b = −0.001, 95% CI = −0.016–0.016) and parental modeling (b = −0.011, 95% CI = −0.029–0.005). Specifically, parent–teen co-decision making was positively associated with adolescents’ self-efficacy for limiting S&J (b = 0.135, 95% CI = 0.087–0.182), but the associations between parental control (b = 0.002, 95% CI = −0.035–0.036) and parental modeling (b = 0.027, 95% CI = −0.011–0.065) and adolescents’ self-efficacy for limiting S&J were not statistically significant. In turn, adolescents’ self-efficacy for limiting S&J predicted less S&J consumption (b = −0.421, 95% CI = −0.514–−0.328). Several direct effects also appear here. After controlling for self-efficacy, parental modeling (b = −0.115, 95% CI = −0.217–−0.013) was still negatively correlated to adolescents’ S&J consumption, whereas parental control (b = 0.190, 95% CI = 0.094–0.286) was positively related to the consumption of S&J. Additionally, parent–teen co-decision making was not associated with the consumption of S&J (b = −0.078, 95% CI = −0.207–0.051) (Figure 3).

## 4. Discussion

Based on Kelmans’ theory of social influence, we considered the three mechanisms of parental influence and examined their relationship with Chinese adolescents’ food consumption, while testing the mediating the effect of self-efficacy in the relationship. We found that all three parental influence factors were positively correlated with adolescents’ F&V consumption, and parental modeling and parent–teen co-decision making were negatively associated with adolescents’ S&J consumption. Parental control was positively linked to the consumption of S&J. Self-efficacy mediated the effects of parental control and parental modeling on adolescents’ consumption of F&V, and the effect of parent–teen co-decision making on S&J consumption.

In agreement with previous studies [23,39,40,41], we found parental control had a positive correlation with adolescents’ consumption of S&J. This result illustrated that parental control that coercively asks their adolescents not to consume too many sugar-sweetened beverages or junk foods may be counterproductive and might have an undesired effect on adolescents’ actual intake. Coercive control may contribute to psychological reactance among adolescents, whose desire for unique and autonomy is evident. Doing the prohibited act or not adapting the promoted behavior is a direct way to restore freedom when it is threatened or eliminated [42]. In addition, the positive association between parental control and adolescents’ consumption of S&J may be in part confounded by peer influence. Notably, in our study, adolescents who reported their friends eat S&J on most days of the week consumed more S&J. Several studies have indicated that peer norms influence adolescents’ consumption behavior [12,13,14]. These could be helpful to explain why parental control leads to the counterproductive effect. Previous research findings on parental control and healthy food consumption are mixed, with results showing both promote and suppresses effects of stronger control on healthy food intake. According to a recent review including 31 studies examined the relationship between parental control and fruit and vegetable intake, three studies reported a negative relationship, 13 suggested a positive relationship, and 17 reported a non-significant relationship with healthy eating [24]. In our analyses, we found parental control has a positive association with increasing Chinese adolescents’ F&V consumption. Future studies are needed to establish a clear causal relation between parental control and adolescents’ healthy food consumption.

Our findings are consistent with previous research noted that parental modeling is consistently, protectively associated with healthy diet [23,24,30,40]. These findings suggest that parents could positively affect adolescents’ eating behavior and food choices by eating more F&V and fewer S&J around adolescents. According to Kelman, identification refers to the process in which human accept an influence by learning or modeling from someone who is respected or admired [21,22]. Social cognitive theory also suggests that modeling refers the process whereby a person learns by observing another person performed behavior [43]. Parental modeling effects take place because adolescents’ own beliefs about nutrition intakes will be influenced by parents’ consumption [44]. Moreover, food availability in the home environment may explain the relationship [35]. When parents choose to eat more F&V and fewer S&J around adolescents, for adolescents, F&V availability in the home environment will increase and S&J availability in the home environment will decrease [38].

Our results indicated that engagement of adolescents in the decision-making process of their own eating plan could potentially facilitate positive effects, including more F&V and less S&J intake. This finding is consistent with prior research that showed parent–teen co-decision making was positively associated with adolescents’ F&V consumption [23,45]. Adolescence is a critical time to form health-related attitudes and behaviors [46]; therefore, involving adolescents in decision-making may give adolescents the opportunity to receive develop their own value systems toward to a sustainable healthy dietary habit.

Our research examined the mediating effect of self-efficacy to the relations between parental influence and adolescents’ eating behavior. As in a prior study, we found adolescents’ self-efficacy for eating F&V was positively related to F&V consumption. We also found parental control and parental modeling were both positively related to increasing adolescents’ self-efficacy toward eating F&V. This is in contrast to prior research that has found that parental control was negatively associated with American adolescents’ self-efficacy toward eating F&V [23]. In addition, our results showed adolescents’ self-efficacy mediated the relationships between parental control and parental modeling on adolescents’ F&V consumption, while also mediating the effect of parent–teen co-decision making on S&J consumption. A prior study suggested that self-efficacy mediated the relations among all three processes of parental influence and both F&V and S&J consumption [23]. These findings suggest that self-efficacy should be explored as viable options for nutrition improvement programs. Additional studies are needed to explore the mediating role of self-efficacy in the relations between three processes of parental influence and adolescents’ dietary choices.

Considering there are more than 166 million adolescents in China, even a small improvement in dietary quality could have significant public health implications [47]. Research has shown that adolescents’ food consumption is strongly influenced by close social connections, which provides opportunities for prevention [48]. The development of effective strategies to improve Chinese adolescents’ eating behaviors requires an understanding of how parents influence adolescents eating behavior [49]. Studies of associations of parental influence and adolescents eating behaviors in Chinese populations are scarce. A nationally longitudinal study reported parental food intake is highly correlated with Chinese children’s food intake [50]. Another study indicated parents should implement control to promote healthy food intake [51]. The present study was the first study to evaluate how perceived parental control, perceived parental modeling, and parent–teen co-decision making influence Chinese adolescents’ dietary behaviors while examining the mediating effect of self-efficacy. Our study provides some behavioral targets that can be used to guide nutrition interventions and education to improve Chinese adolescents’ eating behaviors. Based on our findings, we recommend parents not to exert too much control, especially coercively asking their adolescents not to eat too much unhealth food. Instead, parents should be encouraged to engage adolescents in the decision-making process of their own eating plan. Furthermore, parents could try to provide a positive role model on nutrition intakes. In addition, increasing adolescents’ self-efficacy may contribute to increase the consumption of healthy foods and decrease that of unhealthy foods.

The present study does have some limitations. First, even the sample size of our research is relatively large, the adolescents were recruited from one school, which limits the generalizability of the results. Second, although we have adjusted the data for multiple confounding factors, it is possible that residual factors not captured might modify the relation between parental influence and adolescents’ food consumption. Third, dietary assessment was based on a single 7 day dietary recall, which can be subjected to recall bias and misreporting. Forth, as the present study is a cross-sectional study, we cannot determine causality.

## 5. Conclusions

The present study sheds light on the association between three parental influence factors, F&V and S&J consumption among Chinese adolescents. All three types of parental influence were positively associated with adolescents’ F&V consumption. Parental modeling and parent–teen co-decision making were negatively related to adolescents’ S&J consumption, whereas parental control was positively linked to the consumption of S&J. Adolescents’ self-efficacy contributed to higher F&V consumption and lower S&J consumption. Nutrition interventions in China should focus on increasing parental modeling, parent–teen co-decision making and the adolescents’ self-efficacy.

## Figures and Tables

**Table 1:** Baseline characteristic of participants.

Characteristics ^1^		
Age, years	14.67	1.73
Gender, *n* (%)		
Male	1899	52.82
Female	1696	47.18
Adolescents’ educational level, *n* (%)		
Junior high school	1632	45.40
Senior high school	1963	54.60
Parental education level, *n* (%)		
Illiterate or primary school	232	6.45
Middle school or high school	2414	67.15
College or above	949	26.40
Monthly household income, RMB		
≤5000	461	12.82
5000–10,000	1641	45.65
10,000–20,000	865	24.06
20,000–40,000	360	10.01
≥40,000	268	7.46
Perceived health status, *n* (%)		
Excellent	467	12.99
Very good	743	20.67
Good	1439	40.03
Fair	850	23.64
Poor	96	2.67
Perceived weight status, *n* (%)		
Very underweight	146	4.06
A little underweight	511	14.21
Just right	1058	29.43
A little overweight	1458	40.56
Very overweight	422	11.74
BMI	21.12	4.65
MVPA per week (minutes)	751.05	187.41

^1^ Values are mean (SD), unless otherwise specified.

**Table 2:** Descriptive and correlation matrix among parental influence, self-efficacy, and F&V or S&J consumption.

	Mean	SD	1	2	3	4	5
1. Parental control F&V	3.69	0.92	1				
2. Parental modeling F&V	3.55	1.15	0.45 *	1			
3. Parent–teen co-decision making F&V	3.13	1.23	0.57 *	0.39 *	1		
4. Self-efficacy F&V	3.65	1.16	0.22 *	0.14 *	0.15 *	1	
5. Consumption F&V	3.51	2.69	0.23 *	0.15 *	0.20 *	0.32 *	1
	Mean	SD	6	7	8	9	10
6. Parental control S&J	2.72	1.21	1				
7. Parental modeling S&J	3.54	1.23	0.22 *	1			
8. Parent–teen co-decision making S&J	3.28	1.02	0.34 *	0.50 *	1		
9. Self-efficacy S&J	3.45	1.22	0.48 *	0.11 *	0.15 *	1	
10. Consumption S&J	2.84	3.28	0.05 *	−0.10 *	−0.11 *	−0.24 *	1

* *p* < 0.01. Note: SD = standard deviation.

**Table 3:** Ordinary least-squares regression of adolescents’ fruit and vegetable or sugar-sweetened beverage and junk food consumption.

Variable	b (SE)	95% CI for b	*p* Value
Parental control F&V	0.28 (0.06)	0.16–0.40	<0.001
Parental modeling F&V	0.10 (0.04)	0.01–0.18	0.024
Parent–teen co-decision making F&V	0.20 (0.05)	0.11–0.28	<0.001
Variable	b (SE)	95% CI for b	*p* Value
Parental control S&J	0.19 (0.05)	0.10–0.29	<0.001
Parental modeling S&J	−0.12 (0.05)	−0.22–−0.01	0.026
Parent–teen co-decision making S&J	−0.14 (0.07)	−0.28–−0.01	0.029

Note: adjusted for age, gender, adolescents’ educational level, parental education level, monthly house-hold income, perceived health status, perceived weight status, perceived peer influence, BMI, and physical activity; SE = standard error.

## Data Availability

The data presented in this study are available on request from the corresponding author. The data are not publicly available due to privacy restrictions.

## Supplementary Materials

The following are available online at https://www.mdpi.com/article/10.3390/nu13124454/s1. Table S1. Measures of parental influence, self-efficacy, perceived peer influence, and food consumption.
