# Supplementary material for: Modeling Parental Influence on Food Consumption among Chinese Adolescents through Self-Efficacy: A Path Analysis
Source: Nutrients. 2021 Dec 14;13(12):4454. doi: 10.3390/nu13124454 (PMC8705551; doi:10.3390/nu13124454)
Supplement: Supplementary file 1 [file nutrients-13-04454-s001.zip › nutrients-1458693-supplementary.pdf]

Table S1. Measures of parental influence, self-efficacy, perceived peer influence, and food consumption.

	Items	Response options	Scoring
Parental control F&V	My parent(s) have to make sure that I eat enough fruits and vegetables<br><br>My parent(s) make me eat fruits and vegetables	Strongly disagree = 1,<br>Somewhat disagree = 2,<br>Neither disagree nor agree =3,<br>Somewhat agree =4,<br>Strongly agree =5.	Average score
Parental modeling F&V	My parent(s) try to eat fruits and vegetables when I'm around	Strongly disagree = 1,<br>Somewhat disagree = 2,<br>Neither disagree nor agree =3,<br>Somewhat agree =4,<br>Strongly agree =5.	
Parent-teen co-decision making F&V	My parent(s) try and I decide together how many fruits and vegetables I have to eat	Strongly disagree = 1,<br>Somewhat disagree = 2,<br>Neither disagree nor agree =3,<br>Somewhat agree =4,<br>Strongly agree =5.	
Parental control S&J	My parent(s) have to make sure that I don't drink too much sugar-sweetened beverage or eat too much junk food<br><br>My parent(s) don't buy a lot of sugar-sweetened beverage or junk food for me	Strongly disagree = 1,<br>Somewhat disagree = 2,<br>Neither disagree nor agree =3,<br>Somewhat agree =4,<br>Strongly agree =5.	Average score

Parental modeling S&J	My parent(s) try to avoid eating sugar-sweetened beverage and junk food when I'm around	Strongly disagree = 1,<br>Somewhat disagree = 2,<br>Neither disagree nor agree =3,<br>Somewhat agree =4,<br>Strongly agree =5.
Parent-teen co-decision making S&J	My parent(s) and I decide together how much sugar-sweetened beverage or junk food I can have	Strongly disagree = 1,<br>Somewhat disagree = 2,<br>Neither disagree nor agree =3,<br>Somewhat agree =4,<br>Strongly agree =5.
Self-efficacy F&V	I feel confident in my ability to eat fruits and vegetables every day	Strongly disagree = 1,<br>Somewhat disagree = 2,<br>Neither disagree nor agree =3,<br>Somewhat agree =4,<br>Strongly agree =5.
Self-efficacy S&J	I feel confident in my ability to limit the amount of junk food or sugar-sweetened beverage I eat and drink	Strongly disagree = 1,<br>Somewhat disagree = 2,<br>Neither disagree nor agree =3,<br>Somewhat agree =4,<br>Strongly agree =5.
Perceived peer influence F&V	My friends eat fruits and vegetables most days of the week	Strongly disagree = 1,<br>Somewhat disagree = 2,<br>Neither disagree nor agree =3,<br>Somewhat agree =4,<br>Strongly agree =5.

Perceived peer influence S&J	My friends eat junk food or drink sugary drinks on most days of the week	Strongly disagree = 5,<br>Somewhat disagree = 4,<br>Neither disagree nor agree =3,<br>Somewhat agree =2,<br>Strongly agree =1.	
Consumption F&V	1. During the past 7 days, how many times did you drink 100% pure fruit juice like orange, apple, grape, etc.? Don't count fruit-flavored drinks with added sugar<br>2. During the past 7 days, how many times did you eat fruit like apples, bananas, melon, etc.? Count fresh, frozen, canned and dried fruit. Don't count fruit juices.<br>3. During the past 7 days, how many times did you eat a green salad, with or without other vegetables?<br>4. During the past 7 days, how many times did you eat other non-fried vegetables? Don't count green salad or potatoes.<br>5. During the past 7 days, how many times did you eat refried beans, baked beans, black beans or other cooked beans? Don't count green beans or string beans.<br>6. During the past 7 days, how many times did you eat any other kind of potatoes that aren't fried like baked, boiled, mashed or potatoes used in soups and stews?	Never = 0,<br>1 – 3 times in the past 7 days = 0.29,<br>4 – 6 times in the past 7 days = 0.71,<br>1 time per day = 1,<br>2 times per day = 2,<br>3 or more times per day = 3.	Total score
Consumption S&J	1. During the past 7 days, how many times did you drink sweetened fruit drinks? Don't count 100% pure fruit juice or artificially sweetened or diet drinks.<br>2. During the past 7 days, how many times did you drink regular soda or pop? Don't count diet or zero calorie sodas.<br>3. During the past 7 days, how many times did you drink energy drinks like Red Bull, etc.? These drinks usually have caffeine<br>4. During the past 7 days, how many times did you drink sport drinks?	Never = 0,<br>1 – 3 times in the past 7 days = 0.29,<br>4 – 6 times in the past 7 days = 0.71,<br>1 time per day = 1,	Total score

	5. During the past 7 days, how many times did you eat any type of candy or chocolate? Count candy bars, lollipops/suckers, sour candies, etc. Don’t count sugar-free candy.<br>6. During the past 7 days, how many times did you eat cookies, cakes, cupcakes, doughnuts, brownies, pop-tarts, etc.?<br>7. During the past 7 days, how many times did you eat regular potato chips, corn chips or cheese puffs?<br>8. During the past 7 days, how many times did you eat fired potatoes?<br>9. During the past 7 days, how many times did you eat ice cream or other frozen desserts? Don’t count sugar-free kinds.	2 times per day = 2,<br>3 or more times per day = 3.

Note: F&V = fruit and vegetable. S&J = sugar-sweetened beverage and junk food.
